# Supplementary material for: Assessing soil-transmitted helminths and Schistosoma mansoni infections using parasitological indicators after seven years of preventive chemotherapy among school-age children in Mizan-Aman town
Source: PLoS Negl Trop Dis. 2025 May 8;19(5):e0013058. doi: 10.1371/journal.pntd.0013058 (PMC12061090; doi:10.1371/journal.pntd.0013058)
Supplement: Attachment — Submitted filename: Response to Reviewers.docx [file pntd.0013058.s003.docx]

**Point-by-Point Response to the Reviewers’ comments**

**Manuscript Title:** Assessing the Status of Soil-transmitted Helminths and Schistosoma mansoni infections toward the parasitological indicators set by the elimination program after seven years of preventive chemotherapy among school-age children in Mizan-Aman Town

**Manuscript ID:** PNTD-D-24-01726

We sincerely thank the reviewers for their thoughtful comments and constructive suggestions, as well as the editor for their guidance throughout the review process. These insights have greatly enhanced the quality of our manuscript. Below, we provide **a point-by-point response** to each comment, detailing the revisions made accordingly and the response for the reviewer was included in the manuscript through the track change.

**Reviewer #1: Minor comments**:

- No limitations were indicated which are obvious, from the study design and the uptake of the treatment (coverage and compliance) were not reported

**Response:** Thank you for highlighting the importance of discussing study limitations. We have now included a section addressing the key limitations

- Line 29- there is no large microhaematuria- this is Macrohaematuria, the same for line 30'

**Response**: Sorry I could not find the comment about microhaematuria on the mentioned line

- Lines 50-51: English grammar check.

**Response**: Thank you for your suggestion. We have carefully reviewed and addressed all language issues and have revised these lines to improve grammatical clarity in our manuscript.

- Lines 229-238: Repetition of results.

**Response**: Thank you for your thoughtful suggestion. We would like to clarify that the first paragraph presents the results for soil-transmitted helminths (STH), while the second paragraph focuses on Schistosoma mansoni. However, we have carefully reviewed the section to ensure clarity and avoid any unnecessary repetition

**Reviewer #2: Comments**

- The title could be shortened as follow: “Assessing the Status of Soil- transmitted Helminths and Schistosoma mansoni infections after seven years of preventive chemotherapy among school-age children in Mizan-Aman Town”

**Response:** Thank you for your feedback. My study does not rely on baseline data but assesses infections using parasitological indicators set by the elimination program, focusing on prevalence and intensity of STHs and *S. mansoni* after a year of intervention. WHO guidelines adjust preventive chemotherapy (PC) frequency based on prevalence, with elimination targets aiming for <2% intensity after sustained interventions. To accurately reflect this focus, I revised the title to: 'Assessing Soil-Transmitted Helminths and *S.mansoni* Infections Using Parasitological Indicators After Seven Years of Preventive Chemotherapy Among School-Age Children in Mizan-Aman Town

- The authors speak of seven years of MDA in the study area. It would have been very relevant to have the pre-intervention data to give benefit to this manuscript. Is it possible to have these data in this manuscript? At least those of prevalence and intensities. These data should be incorporated into the MS and the methods adjusted

**Response:** We sincerely appreciate Your insightful comment on pre-intervention data. Unfortunately, such data are unavailable for this study area. However, we assess program progress by comparing current STH and *S. mansoni* prevalence and intensity with elimination program thresholds. Even without baseline data, this approach effectively evaluates the impact of seven years of MDA. WHO recommends using parasitological indicators to track progress in endemic areas lacking baseline data. This widely accepted method provides valuable insights into elimination efforts. We have clarified this in the manuscript and explicitly stated the comparison in the Methods section. Thank you again for your valuable feedback.

- L132-L133: “Children who tested positive for intestinal parasites were referred to local health centers for appropriate treatment.” Did the PZQ and ALB made available in the health centers by the control programme or the government for free?

**Response**: Thank you for your insightful comment. Yes, Praziquantel (PZQ) and Albendazole (ALB) were provided free of charge through the national deworming program. While these medications were supplied by the zonal health unit, we coordinated with the health unit to ensure their availability at local health centers, where positive children received appropriate treatment.

- L142-L143: Please write “The total population of Mizan-Aman town is estimated to be 123,005, of which 63,963 are males as obtained from Mizan-Aman city administration plan commission.” Instead of “The total population of Mizan-Aman town is estimated to be 123,005, of which 63,963 are males and the rest, 59,042, are females as obtained from Mizan-Aman city administration plan commission.”

**Response**: Thank you for your insightful comment, Revised in the manuscript body

- L148: Remove “students”

**Response**: Thank you for your insightful comment, Removed from the manuscript body

- L150: Write, “Sample size calculation and sampling approach” instead of “Sample size determination and sampling techniques”

**Response:** Thank you for your insightful comment, Revised in the manuscript body

- L157-L16: Please revised as follow: “After the selection of schools, the sample size was proportionally allocated to the ten selected schools.” Did you balance the sex in your systematic sampling?

**Response**: Thank you for your insightful comment. In our systematic sampling approach, we selected participants randomly from class rosters without stratifying by sex. As a result, the sex distribution in our sample naturally reflects the composition of the student population.

- L165: what was the structure and content of the questionnaire?

**Response**: Thank you for your valuable feedback. The questionnaire used in this study was structured as a closed-ended survey. It included sections on sociodemographic data, personal factors, and environmental factors to comprehensively capture relevant information from participants.

- What do you mean by “senior laboratory technologists”? did you mean senior laboratory technicians?

**Response**: Thank you for your comment. In this study, 'senior laboratory technologists' refers to senior-degree laboratory personnel with advanced training and responsibilities in laboratory practice. However, if 'senior laboratory technicians' is the more appropriate term in this context, we are happy to revise it for clarity

- L175: When the slides for the other parasites have been read?

**Response**: Thank you for your insightful question. For parasites other than hookworm, we did according to the WHO (2019) SOPs, all other parasites were examined after at least one hour of smear preparation, within a 24-hour period. I revised the writing in the manuscript for clarity

- L193-L200: Please remove this. This information can be display on footnote under tables or figures, L206-L207:

**Response**: Thank you for your insightful comment, Revised in the manuscript body

- Please write “Most of the study participants (411, 66.8%) failed in the age range of 10-14 years with mean age of 10.7.”

**Response**: Thank you for your insightful comment, Revised in the manuscript body

- = L210: “From this STHs infection was 312 (50.7%); 95% CI: (46.8-54.8)”????

**Response**: Thank you for your insightful question, we want to refer from to the percentage of parasitic infections detected, however, I have revised the wording in the manuscript for clarity

- L211: write “the most frequently observed STHs species was *T. trichiura* (48%; 95% CI: 44-52), followed by *A. lumbricoides* (8.3%; 95% CI: 6.2-10.8) and hookworm (1.5%; 95% CI: 0.7-2.8).” Please, revise that through the MS.

**Response**: Thank you for your insightful comment, Revised in the manuscript body

- L218: Please remove “was”

**Response**: Thank you for your insightful comment, Removed from the manuscript body

- L225: remove this “calculated by formula 𝑀&𝐻𝐼 = 𝑀𝑒𝑑𝑒𝑟𝑎𝑡𝑒 𝑖𝑛𝑡𝑒𝑛𝑠𝑖𝑡𝑦+𝐻𝑒𝑎𝑣𝑦 𝑖𝑛𝑡𝑒𝑛𝑠𝑖𝑡𝑦”

**Response**: Thank you for your insightful comment, Removed from the manuscript body

- L 235: “The percent in the table indicates the prevalence within the category of characteristics” remove this

**Response**: Thank you for your insightful comment, Removed from the manuscript body

- Table 3, please remove footnote (L241 and L242)

**Response**: Thank you for your insightful comment, Removed from the manuscript body

- Table 3: write “Number of infected children” instead of “Number of parasites detected”. Note that it is not possible to count parasites with the techniques you used. However, the number of parasites’ eggs can be counted.

**Response**: Thank you for your insightful comment, Revised in the manuscript body

- Please revise Figure 1A by considering the following categories: no infection, mono-infection, bi-infection, three-infection and remove the Figure 1B

**Response**: Thank you for your valuable and constructive feedback. We really agree that Figure 1B does not significantly contribute to the study’s objectives and we have removed it as suggested. In Figure 1A, we want show the overall proportion of the overall multiple infections (dual or triple infections), since it contributes to morbidity among SAC, in addition to the morbidity caused by moderate and heavy infection intensity. Moreover, a high prevalence of multiple infections indicates sustained transmission in the area. We appreciate your thoughtful suggestions and hope this revision aligns with your expectations.

Thank you for your continued support and consideration.

Thank you for your continued support and consideration.

Best regards,

Mitiku Abera
