# Supplementary material for: Assessing soil-transmitted helminths and Schistosoma mansoni infections using parasitological indicators after seven years of preventive chemotherapy among school-age children in Mizan-Aman town
Source: PLoS Negl Trop Dis. 2025 May 8;19(5):e0013058. doi: 10.1371/journal.pntd.0013058 (PMC12061090; doi:10.1371/journal.pntd.0013058)
Supplement: Attachment — Submitted filename: Reviewer Comment (14 1 2025).docx [file pntd.0013058.s002.docx]

Author  : Sánchez-Marqués et al.

Title       : Assessing the Status of Soil-transmitted Helminths and Schistosoma mansoni infections toward the parasitological indicators set by the elimination program after seven years of preventive chemotherapy among school-age children in Mizan-Aman town

Journal      : PLoS Negl Trop Dis

Reference : PNTD-D-24-01726

**General Comment**

The authors are dealing with data, which will be useful for Ethiopia. Please find below comments and suggestions for improvement that the authors could trust considering.

**Title**

= The title could be shorten as follow: “Assessing the Status of Soil-transmitted Helminths and *Schistosoma mansoni* infections after seven years of preventive chemotherapy among school-age children in Mizan-Aman town”

Abstract

= The authors speak of seven years of MDA in the study area. It would have been very relevant to have the pre-intervention data to give benefit to this manuscript. Is it possible to have these data in this manuscript? At least those of prevalence and intensities. These data should be incorporated into the MS and the methods adjusted.

**METHODS**

= L132-L133: “Children who tested positive for

133 intestinal parasites were referred to local health centers for appropriate treatment.” Did the PZQ and ALB made available in the health centers by the control programme or the government for free ?

= L142-L143: Please write “The total population of Mizan-Aman town is estimated to be 123,005, of which 63,963 are males as obtained from Mizan-Aman city administration plan commission.” Instead of “The total population of Mizan-Aman town is estimated to be 123,005, of which 63,963 are males and the rest, 59,042, are females as obtained from Mizan-Aman city administration plan commission.”

= L148: Remove “students”

L150: Write, “Sample size calculation and sampling approach” instead of “Sample size determination and sampling techniques”

L157-L16: Please revised as follow: “After the selection of schools, the sample size was proportionally allocated to

the ten selected schools.”

= Did you balance the sex in your systematic sampling?

= L165: what was the structure and content of the questionnaire?

= What do you mean by “senior laboratory technologists”? did you mean senior laboratory technicians?

L175: When the slides for the other parasites have been read?

= L193-L200: Please remove this. This information can be display on footnote under tables or figures

L206-L207: Please write “Most of the study participants (411, 66.8%) failed in the age range of 10-14 years with mean age of 10.7.”

= L210: “From this STHs infection was 312 (50.7%); 95% CI: (46.8-54.8)”????

L211: write “the most frequently observed STHs species was *T. trichiura* (48%; 95% CI: 44-52), followed by *A. lumbricoides* (8.3%; 95% CI: 6.2-10.8) and hookworm (1.5%; 95% CI: 0.7-2.8).” Please, revise that through the MS.

L218: Please remove “was”

_ L225: remove this “calculated by formula 𝑀&𝐻𝐼 = 𝑀𝑒𝑑𝑒𝑟𝑎𝑡𝑒 𝑖𝑛𝑡𝑒𝑛𝑠𝑖𝑡𝑦+𝐻𝑒𝑎𝑣𝑦 𝑖𝑛𝑡𝑒𝑛𝑠𝑖𝑡𝑦”

= L 235: “The percent in the table indicates the prevalence within the category of characteristics” remove this

= Table 3, please remove footnote (L241 and L242)

= Table 3: write “Number of infected children” instead of “Number of parasites detected”. Note that it is not possible to count parasites with the techniques you used. However, the number of parasites’ eggs can be counted.

= Please revise *Figure 1A by considering the following categories: no infection, mono-infection, bi-infection, three-infection and remove the Figure 1B*
